# Pepper Weevil (Coleoptera: Curculionidae) Preferences for Specific Pepper Cultivars, Plant Parts, Fruit Colors, Fruit Sizes, and Timing

**DOI:** 10.3390/insects7010009

**Published:** 2016-03-04

**Authors:** Dakshina R. Seal, Cliff G. Martin

**Affiliations:** Tropical Research and Education Center (TREC), University of Florida, 18905 SW 280th Street, Homestead, FL 33031, USA; cgm@ufl.edu

**Keywords:** *Anthonomus eugenii*, cultural control, scouting

## Abstract

Peppers (*Capsicum* spp.) are an important crop in the USA, with about 32,000 ha cultivated in 2007, which resulted in $588 million in farm revenue. The pepper weevil, *Anthonomus eugenii* Cano (Coleoptera: Curculionidae), is the most troublesome insect pest of peppers in the southern United States. It is therefore urgent to find different vulnerabilities of pepper cultivars*,* fruit and plants parts, fruit colors and sizes, and timing to infestation by *A. eugenii*. Also relevant is testing whether fruit length and infestation state affect fruit numbers, weights, and proportions of fruit that are infested. Counts of *A. eugenii* adults and marks from oviposition and feeding suggested that *C. chinense* Jacquin “Habanero” was least susceptible, and *C. annuum* L. cultivars “SY” and “SR” were most susceptible*.* Comparison of plant parts and fruit sizes revealed that *A. eugenii* preferred the peduncle, calyx, and top of pepper fruits over the middle, bottom, leaves, or remainder of flowers. *Anthonomus eugenii* does not discriminate between green or yellow fruit color nor vary diurnally in numbers. Based on adult counts, medium to extra-large fruits (≥1.5 cm long) attracted more weevils than small fruits (<1.5 cm). However based on proportions of fruit numbers or fruit weights that were infested, there were no differences between large and small fruits. Choice of pepper cultivar can thus be an important part of an IPM cultural control program designed to combat *A. eugenii* by reduced susceptibility or by synchronous fruit drop of infested fruits. Our results are potentially helpful in developing scouting programs including paying particular attention to the preferred locations of adults and their sites of feeding and oviposition on the fruit. The results also suggested the potential value of spraying when the fruits are still immature to prevent and control infestation.

## 1. Introduction

Two production classes of peppers (*Capsicum* spp.; Solanales: Solanaceae) are produced commercially in the USA: Bell (mild) and Chili (spicy) [1]. In 2007, total pepper production in the USA was about 22,000 ha of Bell and 10,000 ha of Chili peppers with production values of $468 million and $120 million, respectively [1]. The pepper weevil, *Anthonomus eugenii* Cano (Coleoptera: Curculionidae), is the most problematic insect pest of peppers in the southern United States [2]. Although native to Mexico, *A.* *eugenii* is found throughout most of Central America, the Caribbean, and the southern USA from California to Florida, where it became established in 1935 [2,3,4]. According to Speranza *et al.* [5] it also has become established in southern Europe. Because of pepper production outside these areas and international shipments, it also may be found in other pepper-growing regions, such as in Australia, Africa, and Asia.

One generation is completed in 20 to 30 days, but 3 to 5 generations are produced annually [2,3]. The female makes an egg cavity with her mouthparts, deposits a single egg beneath the bud or fruit surface, then covers the cavity containing the egg with a light brown fluid that hardens and darkens [2,6]. A female oviposits 5–7 eggs per day with a mean fecundity of 341 eggs per lifetime [2,3,7]. The egg stage (4.3 days) is followed by the first (1.7 days), second (2.2 days), and third (8.4 days) instars, then the pupa (4.7 days), followed by the adult stage, hence, the duration from egg to adult emergence is 21.3 days [2,3]. Sizes of *A. eugenii* include the egg (0.5 mm long); three instars (1 mm, 1.9 mm, and 3.3 mm, respectively); pupa (~3 mm); and adult (2.0–3.5 mm) [2]. Pupation occurs within a flower or fruit, and upon eclosure from the pupa, the adult escapes from the fruit by making a round exit hole [2]. These data on fecundity, stage duration, and size were based on some of the earliest studies [3,7], which did not specify the temperature or humidity. However, Toapanta *et al.* [6] found that maximum *A. eugenii* fecundity (3.1 eggs/female/days), shortest development time (12.9 days), and minimal mortality were obtained at 30 °C, the temperature for maximum population increase.

To initially find food plants, insects use cues varying from general to host-specific, such as volatiles, then short-range, olfactory, mechanical, and gustatory cues help to verify the suitability of the plant for feeding or oviposition [8,9]. *Anthonomus eugenii* larvae develop only on plants in the Solanaceae, and females oviposit only on *Capsicum* and *Solanum*, though adults also feed on *Physalis*, *Lycopersicon*, *Datura*, *Petunia*, and *Nicotiana* [2,10]. Addesso and McAuslane [9] found that *A. eugenii* females oviposited on all *Capsicum* spp., but only on some *Solanum* spp., for example, not on tomatoes. Among vegetables, *A. eugenii* is most problematic on its namesake and attacks all pepper cultivars (*Capsicum* spp.). However tomatillo, *Physalis philadelphica* Lam., is also moderately susceptible, and eggplants *Solanum melongena* L. grown near peppers are occasionally infested [2]. Among weeds, several species of nightshades support *A. eugenii*, including the common black nightshade *Solanum americanum* Mill. [2,10,11]. These and other nightshade species grow along perimeters of fields previously planted with peppers, and they often serve as alternate hosts when peppers are absent [11,12]. Hence, nightshade weeds should be destroyed before planting the next pepper crop to help prevent infestation.

Pepper plants may become infested before flowering, though oviposition begins only after the plants produce buds and flowers [13]. Plants may become defoliated and fruit production prevented with 30–40 weevils per plant; in severe cases, 70%–90% of the flowers and buds may be infested [13,14]. When suitable pepper fruit are absent, *A. eugenii* may consume stamens and pollen to complete its life cycle [13], and the larvae may complete their development within fallen fruits that do not desiccate. Genung and Ozaki [15] found that all the fallen fruits they examined were infested with *A. eugenii*. Primary damage is caused by larval feeding on seeds and other parts within fruits, which become contaminated with frass, insect parts, and decaying plant tissue often rendering the core brown and moldy [2,3,4,11,16]. Brutton *et al.* [17] noted a direct relationship between the extent of *A. eugenii* damage and the amount of internal mold caused by *Alternaria alternata* (Fr.) Keissler. Secondary fruit damage occurs because of adult feeding and oviposition, which also allows the entry of pathogens [2,3,4,16]. Puncture marks from feeding adults appear as dark specks on the fruits, which are not very damaging, but the fruits are sometimes deformed [2]. Both adult and larval feeding destroy and cause premature abscission of flower buds, flowers, and immature fruit [2,3,4,16,18,19]. On infested fruits, the peduncle turns yellow and the fruit itself turns yellow or red prematurely [2]. Fruit drop is very common and may be the most obvious sign of infestation.

*Anthonomus eugenii* is difficult to control with insecticides once populations are established in the field because the eggs are deposited within flower buds and fruits where larvae and pupae are inaccessible to insecticides or natural enemies [19,20,21]. Scouting is recommended to plan and commence pest management programs and to allow for timing of pesticide applications. Adult population estimates are best obtained by visual examination or by yellow sticky traps [22]. According to Andrews *et al.* [23], an economic threshold recommended when visually scouting for *A. eugenii* is one adult per 100 plants. Action thresholds of one adult per 400 terminal buds [21] or 1% of buds infested also have been suggested [21,24]. However, one 375-cm^2^ trap captures as many *A. eugenii* adults as are detected by inspecting 50 buds [25].

Berdegue *et al.* [4] found that production of pepper fruit began about 40 days after transplanting, and the fruit reached mean sizes above 35 g about 68 days after transplanting. *Anthonomus eugenii* can infest fruit from flowering until the fruit reaches about 35 g [26]. Hence, there was about a 30-d window of susceptibility to *A. eugenii* oviposition on fruits. Insecticides such as Actara^®^ (Thiamethoxam, Syngenta Co., Auckland, New Zealand) [27] and Vydate^®^ (Oxamyl, Dupont Co., Wilmington, DE, USA) [28] are among the most effective for controlling adult *A. eugenii* [11]. However, disadvantages to insecticide use include the expense and toxicity to humans, wildlife, biocontrol agents, and the development of insecticide resistance. Berdegue *et al.* [4] investigated the availability of fruit within pepper lines (synchronous *versus* non-synchronous fruit production) and compared resistant and susceptible lines. They found that the fruit availability more strongly affected *A. eugenii* infestation levels than differences in preferences among pepper lines. Hence, synchronous, high pepper production levels and abscission of infested fruits may help to reduce the time available for *A. eugenii* to infest fruit, permit the removal of dropped fruit, and reduce sorting costs at harvest [4]. These horticultural practices could allow for more cost-effective control of *A. eugenii* while minimizing pesticide use.

To avoid the sole reliance on insecticides to control *A. eugenii*, host plant resistance is an important component of an integrated pest management program and has proven to be effective in managing agricultural pests [29]. Berdegue *et al.* [4] compared 12 commercial pepper cultivars and 23 virus-resistant lines and found that *A. eugenii* adults did not exhibit preferences within or between cultivars or lines suggesting no differences in resistance or susceptibilities. However, because peppers have shown resistance to other insect pests, differences in resistance may exist among other pepper lines not yet tested. In the present study, the first hypothesis was that differences in cultivars, fruit and plant parts, fruit colors and sizes, and timing cause differential susceptibilities of peppers to *A. eugenii*. The second hypothesis was that fruit length and infestation state significantly affect the numbers and weights of fruit and their proportions infested with *A. eugenii*. Pest management recommendations were made based on the results.

## 2. Materials and Methods

Field and laboratory tests were conducted at the Tropical Research and Educational Center (TREC) and at two nearby commercial fields in the Homestead, FL, area. The soil type was Krome gravelly loam (loamy-skeletal, carbonatic, hypothermic, lithic, udorthents) and was well drained, had a PH of 7.4–8.4, was 34%–76% limestone pebbles (≥2 mm diameter), and had low organic matter content (<2%) [30,31]. The tests were performed during two consecutive years with the first test and year on four groups of *Capsicum* spp. including three cultivars, “Hungarian wax”, “Habanero”, “Jalapeño”, and one cultivar group, Bell. The remainder and majority of the tests were in the spring and summer of the second year on nine *Capsicum* spp. cultivars: *C. annuum* “Cayenne”, “Cherry”, “Cubanelle”, “Hungarian”, “Jalapeño”, “Milta”, “SR”, “SY” and *C. chinense* Jacquin “Habanero”.

### 2.1. Capsicum Cultivars Used

Fruits of the Bell group of pepper cultivars have a bell-like, broad shape and are considered “sweet” because they lack capsaicin, a plant secondary compound providing fruits of most other pepper cultivars with a hot taste [32]. The Australian cultivar “Cayenne” produces 6 mm × 76 mm fruits, which change from green to orange to red when mature and require 85 days from planting to harvest [33]. “Cherry” from the USA produces mildly hot 25 mm × 38 mm peppers that turn from green to red, have thick walls, and require 85 days from planting to harvest [33]. “Cubanelle” from the USA produces sweet, 64 mm × 140 mm peppers that change from light green to yellow to orange to red and require 65 days from planting to harvest [33]. “Habanero” produces very hot, wrinkled, 32 mm × 51 mm fruits that ripen from dark green to orange and require 75 days from planting to harvest for green fruits, or 100 days for orange fruits [34]. “Hungarian” produces hot, 200-mm-long fruit that change from light green to yellow to red when mature and require 70 days from planting to harvest [33]. “Hungarian wax” is very similar and closely related to “Hungarian”, and it produces hot, 130-mm-long fruit that change from yellow to red when mature and require 70 days from planting to harvest [35]. “Milta” is in the “Jalapeño” group and was derived from that cultivar; it produces 25 mm × 64 mm, thick-walled fruit that taper to a blunt tip, ripen from green to red, and require 60–65 days from planting to harvest [36]. “SR”, a sweet bell pepper, produces thick-walled, 75 mm × 90 mm fruit, which ripen to orange or purple and require 50–60 days from flowering to harvest [37]. “SY” is an F-1 hybrid and produces hot 70-mm-long peppers that mature moderately early and have good disease resistance [38]. All cultivars of *C. annuum* and *C. chinense* have green stems, green leaves, white flowers, are upright, and 40 to 90 cm tall when mature. In tests 1, 2, 3, and 6, varieties were compared based on numbers of adults or puncture marks and oviposition plugs per fruit. Fruit sizes varied between cultivars or groups of cultivars; hence, the product of fruit length × width (“silhouette area”) was estimated using the above fruit dimensions and placed into the tables to show relative fruit sizes along with results comparing the taxa.

### 2.2. Collection of A. eugenii Adults

Sufficient numbers of fallen fruits *(ca.* 1000) with an indication of *A. eugenii* infestation were collected from “Jalapeno” pepper fields at TREC to perform the tests. The fruits were washed with tap water and air dried to remove excess water from the surfaces. A layer of the dried fruits were placed within a wooden cubicle (30 cm × 30 cm × 30 cm) in the laboratory to facilitate adult emergence and to avoid decomposition, and they were checked daily to collect adults. Hence, adults used in the laboratory tests were 0–24 h old with a male:female sex-ratio of about 50:50. The 6 tests included Test 5 in the first year, Tests 1–4 and 6 in the second year, and eastern standard time (EST) in the USA was the local time for all the tests. Environmental conditions in the initial adult emergence cubicle and in Tests 1–4 (laboratory tests) were maintained at 28 ± 2 °C, 72% ± 3% RH, and at a 14:10 L:D period, whereas tests 5–6 (field tests) were performed under ambient field conditions.

### 2.3. Preference of Adult A. eugenii for Different Pepper Cultivars, Fruit and Plant Parts, Fruit Colors and Sizes, and Timing (Tests 1–4)

The studies were conducted in a laboratory using three observation cages each housing one or more replications depending on the test and data collected. The cages were rectangular (91 cm × 58 cm area) with the tops of the shorter sides slanting so that the longer sides were 62 cm and 36 cm high on the rear and front, respectively. The cages had wooden frames, glass tops, and 26%–38% of the area of the two longer sides was covered with cloth for ventilation. Each cage contained 2 fruits for each of the 8 cultivars, hence 16 total fruits that were 7-to-9-days old. All fruits were suspended from the opposing shorter sidewalls using four 1-m-long strings; 18 cm separated the centers of four fruits per string from each other and from each sidewall. Strings were elevated about 30 cm above the cage floor, and 12 cm separated adjacent strings from each other and from the longer sidewalls. Four 1.5 cm × 6 cm glass vials were added per cage, one at each corner, and each contained 10% sugar solution with a cotton wick in the center, which was soaked with vial contents to provide additional nourishment for *A. eugenii* adults. After being gathered from the laboratory colony, 100 to 150 *A. eugenii* adults (0–24 h old) were released at the center of each cage. The experimental period for Tests 1–4 collectively spanned 11 days from 26 March to 5 April: Test 1 was 10 days (26 March to 4 April); Test 2, 3 days (2–4 April); Test 3, 1 day (5 April); and Test 4, 1 day (3 April). Five factors were compared in the analyses: plant parts (mainly fruit), fruit sizes, fruit colors, pepper cultivars, and times of observation. When one factor was compared, data from the other factors were usually pooled to form extra replications. For example, when comparing the five plant parts for each of the eight cultivars, the number of adult weevils found at each of 14 observation times served as a replication (Table 1).

#### 2.3.1. Test 1. Preference of Adult *A. eugenii* for Different Pepper Cultivars, Parts of Pepper Fruit, and Observation Times Based on Counts of Adults (Days 1–10 of 11)

Numbers of adult *A. eugenii* found on each fruit were recorded 20 times with each recording 2–65 h apart and varying from 07:00 to 18:30 EST each day. For 14 of these observations, numbers of adults were also noted at each of five locations including the peduncle, calyx, and the top, middle, and bottom thirds of fruit. The procedure was performed in three cages simultaneously during the 9 days (10-day-spanning) test, and although there were initially 3 replications, because of pooling, most analyses and results involved 6 or more replications. Data were divided into two groups each with a separate initial statistical analysis: individual parts (Test 1a–c) and sum of parts for each cultivar (Test 1d). Individual parts included the peduncle, calyx, and the top, middle, and bottom thirds of fruit. For individual parts, a 3-way factorial (8 cultivars × 5 plant parts × 14 observation times) was initially performed to determine if there were interactions between any of the factors and to find their effects on resulting numbers of adult *A. eugenii*. Hence, there were the following three two-way factorials: cultivar × plant part with 8 cultivars, 5 plant parts, and 14 observation times (replications) (Test 1a); cultivar × observation time with 8 cultivars, 14 observation times, and 5 plant parts (replications) (Test 1b); and plant part × observation time with 5 plant parts, 14 observation times, and 8 cultivars (replications) (Test 1c). For the sum of plant parts (Test 1d), two one-way ANOVAs were initially performed including one with 8 cultivars and 20 observation times (replications) and the other with 20 observation times and 8 cultivars (replications).

#### 2.3.2. Test 2. Preference of Adult *A. eugenii* for Different Pepper Cultivars and Different Parts of Pepper Fruit Based on Numbers of Puncture Marks and Oviposition Plugs (Days 8–10 of 11)

All data were taken using fruits, insects, and cages from Test 1 days 8–10 (2–4 April). Test 2 surveyed puncture marks and oviposition plugs made by adults, which were simultaneously counted in Test 1. Each cultivar had two fruits, and to collect data, each fruit was visually divided into the peduncle, calyx, and the top, middle, and bottom thirds. Puncture marks resulting from adult feeding and oviposition on fruit were observed using a 10× hand lens. Three counts (one per cage) of puncture marks and oviposition plugs were taken for each part of two fruits resulting in 6 replications per plant part per pepper cultivar, and the data were total counts for the 3-day test period. Data were divided into two groups each with a separate initial statistical analysis: individual parts (Test 2a) and sum of parts (Test 2b). For individual parts, a factorial was initially performed (5 plant parts × 8 cultivars with 6 replications) to determine interaction, and for the sum of parts, a one-way ANOVA was performed with 8 cultivars and 6 replications.

#### 2.3.3. Test 3. Effects of Fruit Color, Location on Fruit, and Timing on the Numbers of Adult *A. eugenii* (Day 11 of 11)

Fruit colors were green and yellow and estimated by casual visual inspection. Locations on fruit included the peduncle, calyx, and the top, middle, and bottom thirds of fruit, and observation times were 3:00 PM, 5:00 PM, and 7:00 PM EST. Large versus medium-sized fruit were also noted but pooled in all analyses. The test was performed using the foregoing three observation cages on a single day. A three-way factorial was initially performed (three two-way factorials) involving 2 colors, 5 plant parts, 3 observation times, and initially 3 replications. The initial three two-way factorials were Test 3a (2 colors × 5 plant parts, 9 replications), Test 3b (2 colors × 3 observation times, 15 replications), and Test 3c (5 plant parts × 3 observation times, 6 replications).

#### 2.3.4. Test 4. Preference of Adult *A. eugenii* for Different Fruit (and Other) Plant Parts, Fruit Sizes, and Observation Times on Pepper Plants (Day 9 of 11)

Using the previously described 3 observation cages, numbers of adult *A. eugenii* were counted on 4–21 different locations on pepper plants (mostly on fruit), at 8 observation times from 6 AM to 8 PM EST on a single day (3 April). There were four data sets each with a separate statistical analysis beginning with a factorial of 4, 6, 7, or 21 plant parts × 8 observation times and initially 3 replications. The data set with 21 plant parts did not include subtotals for parts, while the sets with 4, 6, or 7 parts included both individual plant parts and parts grouped into selected subtotals. Groups for fruit length included small (<1.5 cm long), medium (1.5–3.4 cm), large (3.5–6.9 cm), and extra-large (≥7.0 cm) fruits. The 21 plant parts (Test 4a) included 5 parts each with medium, large, and extra-large fruit (peduncle, calyx, and the top, middle, and bottom thirds of fruit) resulting in 15 plant parts. For small fruits, however, only peduncles and calyxes (2 parts) were examined. The remaining 4 of 21 plant parts included the peduncles, calyxes, remainder of flowers (mainly corollas), and whole leaves. The analysis for 7 plant parts × 8 observation times (Test 4b) considered the following plant parts and subtotals thereof: leaf, corolla, peduncle, calyx, and the top, middle, and bottom thirds of fruit. Using plant parts grouped to form an initial factorial of 6 parts × 8 observation times (Test 4c), the following 6 parts were considered: flowers, leaves, and small, medium, large, and extra-large fruit. Finally, a factorial was performed with 4 fruit sizes (small, medium, large, and extra-large fruit) × 8 observation times (Test 4d). It included the fruit totals (top, middle, and bottom excluding peduncle and calyx) at each observation time.

### 2.4. Test 5. Effects of Pepper Cultivars and Cultivar Groups on Numbers of A. eugenii Larvae within Fruits

Two separate commercial pepper fields were employed each with four pepper cultivar groups planted separately in adjoining plots: “Hungarian wax”, “Habanero”, “Jalapeño”, and Bell. Each pepper plot was 46 m × 10 m and consisted of 6 beds each 46 m × 1 m, raised 15 cm, and with adjacent bed centers separated by 0.9 m. Irrigation was provided by two drip tapes (T-systems, DripWorks Co., Willits, CA, USA), which were placed on the soil surface with one tape on each side and parallel to each bed and 30 cm from its center. Beds were subsequently covered with 1-mil, black-on-white, polyethylene mulch (Grower’s Solution Co., Cookeville, TN, USA) with the white side facing upward for weed control and to help moderate the soil temperature and moisture. One pepper plant was transplanted into a circular, 10-cm-diam hole in the plastic and 3 cm deep in the soil with holes 40 cm apart in the center of each row. Later, when the crop was fruiting, five plants were randomly sampled per replication, and all fruits for each sample plant were harvested and placed into a plastic container. Each container had a plastic lid with about half its area composed of screen mesh to allow ventilation while preventing the escape of adults, and the fruits were covered with two layers of paper towels to absorb excess moisture. In the laboratory, fruits were dissected and *A. eugenii* larvae were removed, counted, and their numbers were totaled by adding the counts from all the fruits of each sample plant. There were 4 replications (plots) for each treatment in Test 5.

### 2.5. Test 6. Effects of Fruit Length and Infestation State (Infested vs. Non-Infested) on Fruit Numbers and Weights and on the Proportions of Fruit Numbers and Weights that were Infested

The same pepper fields and methods described for Test 5 (larvae within fruit) were used. “Milta” (Test 6a) was planted in one field, and the other field had three adjoining plots, two with “Hungarian” (Tests 6b, c), and one with “Cubanelle” (Test 6d). Similar to the previous test for larvae within fruit, for each replication of the present test, five plants were randomly selected with all fruit per plant harvested and placed into a plastic bag. Upon inspection of each fruit, the following were recorded: infestation state (infested or non-infested), and the quantity, length, weight, and resulting size class of fruits. A fruit was considered infested if it had the characteristic exit holes of adult *A. eugenii* and/or a yellow calyx. The following fruit lengths were used to determine the size classes for “Milta” (Test 6a): small (0–2.0 cm), medium (2.1–4.0 cm), and large (≥4.1 cm). “Hungarian” (Tests 6b,c) fruit sizes were small (0–4.0 cm), medium (4.1–6.0 cm), and large (≥6.1 cm). “Cubanelle” (Test 6d) fruits were small (0–6.0 cm), medium (6.1–10.0 cm), or large (≥10.1 cm). There were 9 bags (9 replications) for Test 6a, 8 replications each for Tests 6b and 6c, and 4 replications for Test 6d. Within each of the four data sets (Tests 6a–d), a one-way ANOVA was used to compare the 3 treatments (size classes of fruit).

## 3. Results and Discussion

### 3.1. Results

Using fruit dimensions from Reimer Seeds [33], Johnny Seeds [34], Newcomb [36], and Made-in-china.com [37,38], the product of fruit length × width (“silhouette area”) allowed the eight cultivars compared to be ranked from highest to lowest as follows for Tests 1 and 2 (Table 1, Table 2 and Table 3): “Cubanelle” > “SR” > “Hungarian” > “SY” > “Jalapeño” > “Habanero” > “Cherry” > “Cayenne”. Similarly, for Test 5 (Table 6), the product of fruit length × width allowed the four taxa to be ranked from highest to lowest as follows: Bell > “Hungarian wax” > “Jalapeño” > “Habanero” [32,34,35,36].

#### 3.1.1. Preference of Adult *A. eugenii* for Different Pepper Cultivars, Parts of Pepper Fruit, and Observation Times Based on Counts of Adults (Test 1)

For numbers of adult *A. eugenii* found in Test 1a, there was a significant interaction (*p* ≤ 0.05) between cultivar and plant part (*F* = 6.11, df = 28, *p* < 0.0001). Significant differences in adult counts were found comparing parts within cultivars for “Cherry”, “Cubanelle”, “Hungarian”, “SR”, and “SY”, but differences were not significant within “Cayenne”, “Habanero”, or “Jalapeño” (Table 1A). “Cherry” yielded significantly more adults on the calyx than on the top thirds of fruit; in turn, the peduncle, calyx, and top thirds each led to significantly more adults than the middle or bottom. “Cubanelle” had significantly more adults on the calyx, peduncle, and top third of the fruit than on the bottom. On “Hungarian”, significantly more adults were found on the peduncle than on the calyx, top, or bottom thirds of fruit, but significantly fewer were on the middle than on any other plant part. “SR” yielded significantly more adults on the calyx and top thirds of fruit than on the peduncle, whereas the middle and bottom thirds each had significantly fewer adults than the other three plant parts. Similarly, “SY” yielded significantly more adults on the top thirds of fruit than on any other plant part and significantly more on the calyx than on the peduncle or bottom. There were significant differences in numbers of *A. eugenii* adults among the eight cultivars for each of the five plant parts in Test 1a (Table 1B). Considering peduncles, “SY” led to significantly more adults than all the other cultivars except for “SR”. In addition, “SY” and “SR” each had significantly more adults on peduncles than all other cultivars except “Hungarian”. In turn, peduncles of “SY”, “SR”, and “Hungarian” each yielded significantly more adults than all the other cultivars except for “Cayenne”, which had significantly more than “Habanero” and “Jalapeño”, the two cultivars with the fewest adults on peduncles. On calyxes, “SY” and “SR” each had significantly more adults than all the other cultivars. “Cayenne”, “Cherry”, and “Cubanelle” each had significantly more adults on calyxes than “Jalapeño” and “Habanero”, the two cultivars with the fewest. On the top third of pepper fruit, “SY” had significantly more adults than all the other cultivars, and “SR” had significantly more than all other cultivars except “SY”. “Cayenne”, “Cubanelle”, and “Hungarian” each had significantly fewer adults than “SR” or “SY”, but significantly more than on “Habanero”, which had the fewest adults per top third. On the pepper fruit middle third, “SY” had significantly more adults than all other cultivars, and “SR” had significantly more than “Cherry”, “Cubanelle”, “Habanero”, or “Hungarian”. “Hungarian” had the lowest number of adult *A. eugenii* numerically and was significantly lower than “SY”, “SR”, “Cayenne”, or “Jalapeño” for the middle third of pepper fruit. Considering the bottom third, “SY” had significantly more adults than all the other cultivars, and of the remaining cultivars, “SR” and “Cayenne” each had significantly more than all the others except “Hungarian”. On the bottom third, “Cherry”, “Cubanelle”, “Habanero”, and “Jalapeño” each had the fewest adults statistically with “Habanero” also having the fewest numerically.

Results of Test 1b–d (Table 2) for numbers of adult *A. eugenii* indicated there were no significant interactions (*p* > 0.05) for cultivar and observation time with plant parts considered separately (Table 2A), cultivar and observation time with plant parts summed (Table 2B), or plant parts and observation time (Table 2C). For cultivar and observation time with plant parts considered separately, results were insignificant comparing observation times with cultivars pooled, but were significant comparing cultivars with observation times pooled (Table 2A). Considering cultivars with plant parts separate and with observation times pooled (Table 2A), “SY” had significantly more adults than all the other cultivars, and “SR” had significantly more than all the other cultivars except for “SY”. Here, “Cayenne”, “Cubanelle”, and “Hungarian”, each had significantly fewer adults than “SR” or “SY”, but significantly more than “Jalapeño” or “Habanero”. “Habanero” had the fewest *A. eugenii* adults numerically and was statistically lower than all the other cultivars except for “Jalapeño” (Table 2A). For cultivar and observation time with plant parts summed, there was no significant difference in the number of adults comparing observation times with cultivars pooled, but there was a significant difference comparing cultivars with observation times pooled (Table 2B). “SY” had significantly more adult *A. eugenii* than all the other cultivars, and “SR” had significantly more than all the remaining cultivars other than “SY”. “Cayenne”, “Cubanelle”, and “Hungarian” each had significantly fewer adults than “SR” or “SY”, but significantly more than “Habanero”. “Habanero” had the fewest adults numerically with significantly fewer than all the other cultivars except “Cherry” or “Jalapeño”. Results for plant part and observation time were insignificant comparing observation times with plant parts pooled, but were significant for plant parts with observation times pooled (Table 2C). The top third of fruit was numerically highest with significantly more adults than the peduncle, middle, or bottom thirds of fruit, and the latter two yielded the fewest adults statistically.

#### 3.1.2. Preference of Adult *A. eugenii* for Different Pepper Cultivars and Different Parts of Pepper Fruit Based on Numbers of Puncture Marks and Oviposition Plugs (Test 2)

There was a significant interaction (*p* > 0.05) between cultivar and plant part based on counts of puncture marks and oviposition plugs (*F* = 3.88, df = 28, *p* < 0.0001). There were significant differences in numbers of puncture marks comparing parts within cultivars for “Cayenne”, “Cherry”, “Cubanelle”, “SR”, and “SY”, but differences were not significant within “Habanero”, “Hungarian”, or “Jalapeño” (Table 3A). “Cayenne” had significantly more adult *A. eugenii* puncture marks on the bottom third of fruit than on the peduncle, calyx, or middle third, and significantly more on the top than on the middle third, which was numerically lowest. On “Cherry”, significantly more puncture marks were found on the peduncle than on any other plant part, and significantly more were on the calyx than on the top, middle, or bottom thirds of fruit. “Cubanelle” and “SR” each had significantly more puncture marks on the peduncle than on the calyx, which had significantly more than on the top, middle, or bottom thirds of fruit. The peduncle and calyx of “SY” each yielded significantly more puncture marks than the bottom or middle thirds. The “SY” middle had the fewest puncture marks numerically with significantly fewer than the peduncle, calyx, or top thirds of fruit (Table 3A).

In the fruit middle section, there were no significant differences among the pepper cultivars in numbers of adult puncture marks, but every other plant part showed significant variation (Table 3B). On peduncles, “Cherry”, “Cubanelle”, and “SR” each yielded significantly more adult puncture marks than “Hungarian”, “Habanero”, or “Cayenne”. “Jalapeño” and “SY” each led to significantly more than “Cayenne” or “Habanero”, which had the fewest. Among calyxes, “SY” had significantly more adult puncture marks than all the other cultivars, and “Cherry” and “Cubanelle” each had significantly more than “Cayenne” or “Habanero”. “SY”, “SR”, “Jalapeño”, “Cherry”, and “Cubanelle” each had significantly more than “Habanero”, which yielded the fewest puncture marks numerically per calyx. On the top third of fruit, “SY” had significantly more puncture marks than “SR”, “Cherry”, “Cubanelle”, or “Habanero”. On the bottom third of fruit, “Cayenne” had significantly more puncture marks than all other cultivars, and among the remaining cultivars, “Hungarian” led to significantly more than “Cherry”, “Cubanelle”, “Jalapeño”, or “SR” (Table 3B). There were significant differences among cultivars in numbers of adult puncture marks when considering totals of the five plant parts (Table 3C). “SY” yielded significantly more puncture marks than all the other cultivars, while “Habanero” had significantly fewer than all the other cultivars (Table 3C).

#### 3.1.3. Effects of Fruit Color, on-Fruit Location (Part), and Timing on the Number of Adults (Test 3)

Based on numbers of adult *A. eugenii, t*here were no significant interactions in any of the three factorials, fruit color × plant part, fruit color × observation time, or plant part × observation time (*p* > 0.05). Results for color and plant part were insignificant comparing fruit colors with plant parts pooled, but were significant comparing plant parts with colors pooled (Table 4A). The top thirds of the fruit yielded significantly more adult *A. eugenii* than the peduncle, which had significantly more than the calyx, middle, or bottom of the fruit. The bottom was numerically the lowest and yielded significantly fewer adults than the peduncle, calyx, or top third of fruit. For color and observation time, results were insignificant comparing fruit colors with observation times pooled or comparing observation times with fruit colors pooled. Considering plant parts and observation times, there were no significant differences among observation times with plant parts pooled, but significant differences were found among plant parts with observation times pooled (Table 4B). Results for plant parts with colors pooled (Table 4A) were therefore identical to those of plant parts with observation times pooled (Table 4B). 

#### 3.1.4. Preference of Adult *A. eugenii* for Different Pepper Fruit Sizes, Plant and Fruit Parts, and Observation Times (Test 4)

There were no significant interactions (*p* > 0.05) between part plant and observation time for numbers of adult *A. eugenii* in any of the four factorials. These included 21 plant parts without totals, 7 plant parts with pooled fruit sizes, 6 plant parts including leaves, flowers, and four fruit sizes, or only four fruit sizes. Test 4a had 21 plant parts with no parts grouped into totals, and it had insignificant results comparing observation times with plant parts pooled, but significant results comparing plant parts with observation times pooled (Table 5A). Here, the top third of extra-large fruit resulted in the most adult *A. eugenii* numerically with significantly more than all the other 20 part-size groups except for the top third of large fruit. On the other hand, the bottom third of extra-large fruit was numerically lowest and significantly lower than all the other groups except for leaves; peduncles, corollas, and calyxes of flowers; peduncles and middle thirds of large fruit; and calyxes and peduncles of small fruit.

Considering numbers of adults found on 7 plant parts including pooled fruit sizes, results were insignificant comparing observation times with plant parts pooled, but were significant comparing plant parts with observation times pooled (Table 5B). The top third of fruit was numerically highest in numbers of adults and significantly higher than all the remaining part groups. The calyx, peduncle, middle, and bottom thirds of fruit all had significantly fewer adults than the top third of fruit but significantly more than the leaves. Leaves were numerically lowest with significantly fewer adults than all the other part groups except for flower corollas (Table 5B). Grouping the data into 6 plant parts × 8 observation times yielded insignificant results comparing observation times with plant parts pooled, but significant results comparing plant parts with observation times pooled (Table 5C). With the 6 plant-part groups, medium-sized, large, and extra-large fruit each led to significantly more adult *A. eugenii* than leaves, flowers, or small fruit. Small fruit yielded significantly more adults than flowers, which provided significantly more than leaves, which had the fewest (Table 5C). When only 4 fruit sizes were considered, the results were insignificant comparing observation times with fruit sizes pooled, but were significant comparing fruit sizes with observation times pooled (Table 5D). Extra-large, large, and medium-sized fruits each yielded significantly more adult *A. eugenii* than small fruits. 

### 3.2. Effects of Fruit of Selected Pepper Varietal Groups on Numbers of A. eugenii Larvae per Plant (Test 5)

The Bell pepper group led to significantly more larvae per plant than the “Hungarian wax” group in Field 1 (Table 6). In Field 2, the “Jalapeño” group had significantly more larvae than the “Hungarian wax” or “Habanero” groups, each with significantly fewer than the Bell or “Jalapeño” groups.

### 3.3. Effects of Infestation State (Infested or Non-Infested) and Fruit Length on Fruit Numbers, Fruit Weights, and the Proportions of Fruit Numbers or Weights that were Infested (Test 6)

For “Milta” (Table 7A), there were no significant differences among fruit-length groups in infested fruit numbers or weights, or in the proportions of fruit numbers or weights that were infested. However, total numbers of fruit and total fruit weights were each significantly different among fruit sizes. Considering total numbers of fruit, there were significantly more medium or large than small fruit. Total fruit weights were significantly heavier for large than for medium fruits, which were significantly heavier than small fruits (Table 7A).

“Hungarian” (Table 7B) yielded no significant differences between fruit sizes in the proportions of fruit numbers or fruit weights that were infested. However, there were significant differences in total fruit numbers and weights, and in the numbers and weights of fruit that were infested. Large fruits were significantly greater than medium or small fruits in total fruit numbers and weights, and in fruit weights that were infested (Table 7B). For numbers of infested fruit, there were significantly more large than small fruit.

Another test with “Hungarian” (Table 7C) resulted in no significant differences among fruit sizes in the proportions of fruit numbers or of fruit weights that were infested or in the numbers of infested fruit. However, there were significant differences among the size classes in total fruit numbers, total fruit weights, and in weights of infested fruit. For total numbers and weights of fruit, there were significantly more and significantly heavier large than medium-sized fruits and significantly more and heavier medium than small fruits (Table 7C). Large fruits yielded significantly heavier infested fruits than small fruits.

“Cubanelle” (Table 7D) yielded no significant differences in the proportions of fruit numbers or weights that were infested or in the numbers or weights of infested fruit. However, significant differences occurred among size classes in total numbers of fruit and in total fruit weights. There were significantly more medium or large than small fruit for “Cubanelle” total fruit numbers. Total fruit weights were significantly heavier for large than medium-sized fruits, which were significantly heavier than small fruits (Table 7D).

### 3.4. Discussion

The two initial field tests during the first year involved counting numbers of *A. eugenii* larvae within fruit of four pepper cultivars. More larvae were found per Bell pepper fruit than per fruit of “Hungarian wax”, which suggested that adults were more attracted to Bell peppers. However, the apparently larger cross-sectional fruit area (length × width) of Bell than “Hungarian wax” (80 *vs.* 30 cm^2^) suggests that Bell may have lured more weevils by having larger fruit. “Habanero” had the fewest larvae and thus appeared to be least preferred in one of two tests, though it had the smallest cross-sectional fruit area numerically. The initial test in the second year found that plant parts from “SY” and “SR” generally attracted larger numbers of adult *A. eugenii* than parts from the other six cultivars. Two additional comparisons of cultivars with observation times pooled yielded similar results: “SY” and “SR” attracted the most adults and “Jalapeño” and “Habanero” the fewest. When considering cultivars within individual plant parts or with plant parts summed, similar results were found based on varying numbers of marks from adult oviposition and puncture. With exception of the middle and bottom thirds of fruit, “SY” had among the highest numbers of puncture marks per plant part and “Habanero” had among the fewest. One of the two tests (two analyses) for varietal preference from the first year and two tests (four analyses) from the second year provided similar results: “SY” was usually most preferred numerically and “Habanero” least preferred with statistical differences between them in 11 of 13 final ANOVAs that included both cultivars. Thus, our field and laboratory experiments showed that *A. eugenii* preferred certain cultivars over others with “SY” and “SR” most preferred and “Habanero” least preferred. Seal and Bondari [13] found that “Hot Cherry” and “Habanero” each had consistently lower infestation levels than nine other cultivars including “Jalapeño”, which was moderately resistant. Our results also suggest that “Habanero” has a relatively low susceptibility or possibly high repellence or antifedence to *A. eugenii* infestation. “Habanero” may therefore produce higher yields using less pesticides and hence serve as a potentially good choice for management by varietal resistance. “Habanero” is a cultivar of *C. chinense,* which was apparently more resistant (or less susceptible) than *C. annuum* to *A. eugenii* in the present study. The other seven test cultivars were derived from *C. annuum.* Although fruit-size differences among the varieties were not compared statistically, “SY” fruit areas were about 20 cm^2^ (4th highest of 8 varieties), “SR” was 2nd highest (72 cm^2^), and “Habanero” was third lowest (15 cm^2^). In addition, the limited number of larvae (usually 1–2) found per infested fruit, which generally appear able support many more larvae, suggests that a marking pheromone limits the number of oviposition events and eggs laid per fruit. Larger fruits may allow for more eggs than small fruits because of the greater distances between sites of adult feeding and oviposition, which may be marked with the pheromone. Hence, smaller fruits may tend to have fewer adults, puncture marks, and oviposition plugs than larger fruits, and fruit size differences may have affected our results. Nonetheless, Seal and Bondari [13] concluded that some cultivars have a stronger natural resistance to *A. eugenii* than others, and plant breeders may be able to enhance the resistance.

In addition, some pepper lines have fruit production that is more synchronous and concentrated than others. They readily shed fruit infested with *A. eugenii*, which helps to reduce the damage compared with lines that shed fewer fruits [4]. Lines with synchronous, concentrated fruit production had a reduced period of susceptibility to attack by *A. eugenii* [4]. In addition to reducing pesticide use because of fewer *A. eugenii* per pepper plant, employing less-susceptible pepper cultivars may enhance the effectiveness of removing dropped, infested fruits as a cultural technique [4]. Choosing pepper cultivars for greater resistance, tolerance, or reduced susceptibility to *A. eugenii*, in addition to synchronous, concentrated fruit production, can therefore serve as effective cultural components of an IPM program.

Based on counts of *A. eugenii* adults, similar results were found when comparing parts within cultivars (from a factorial with interaction) and when comparing parts with observation times pooled (from a factorial with no-interaction). More adults were generally attracted to the calyx, peduncle, or top third of pepper fruit than to the middle or bottom. Based on numbers of puncture marks and oviposition plugs per plant part from most cultivars with variation, the calyx and peduncle each were preferred over the top, middle, or bottom thirds of fruit. In a third test, no-interaction factorial results for color × part and part × observation time were identical because only plant-part differences were significant and either colors or observation times were pooled. Here, peduncles and top thirds of fruit yielded higher numbers of *A. eugenii* than calyxes, middle, or bottom thirds with the top third most preferred and bottom third least preferred. The fourth test yielded four factorial analyses with no-interaction results: one analysis was for 21 plant parts that were compared individually and the other three were for 7, 6, or 4 plant parts or fruit sizes with some or all the plant parts grouped. Medium, large, and extra-large fruit were preferred over small fruit, flowers, and leaves. The top thirds of fruits generally were most preferred with the leaves least preferred. Overall, among the four tests comparing plant parts and/or fruit sizes, *A. eugenii* seemed to prefer the top, peduncle, and calyx over the middle and bottom of pepper fruits. Similarly, Toapanta *et al.* [6] found that regardless of temperature (15–33 °C), *A. eugenii* produced more feeding punctures on the fruit top than on the middle or bottom, and females deposited more eggs on the top and on or near the calyx than on the middle or bottom. Of the eggs oviposited on pepper fruit, 63%–78% were at the top, 14%–25% at the middle, and 4%–18% at the bottom, but unlike in our findings, *A. eugenii* preferred smaller over larger fruit sizes [6]. The peduncle and calyx are initially formed as part of the flower and thus precede the top, middle, and bottom of pepper fruits in development. Thus, control measures may be most effective when plants are flowering and fruits are immature, which is when applied insecticides may be the most preventative.

Boll weevils *Anthonomus grandis grandis* Boheman (Coleoptera: Curculionidae) on cotton *Gossypium hirsutum* L. preferred the flower squares (buds) over bolls (fruit) for oviposition and feeding [39,40]. Egg production and survival to adulthood in laboratory petri dishes and adult feeding and oviposition in the field were each lower on small (1–3 mm) than on large (5.5–8 mm diameter) buds [41,42]. Reproductive portions of large buds provided enough food for *A. grandis* larvae to complete development resulting in greater fecundity than smaller buds or fruit, although they fed less often on large buds than on young fruit (5–10 days old) [43,44]. Overall, feeding on reproductive portions of fruiting bodies promoted more egg production than rinds, and the buds were nutritionally superior to fruit [44]. *Anthonomus* *grandis* and *A.* *eugenii* are closely related species with similar life cycles. Hence, *A. eugenii* may also choose the peduncle, calyx, and top third of fruit over the middle and bottom third, other flower parts, and leaves because of nutritional superiority, fecundity, or fitness advantages.

Green *versus* yellow fruit colors were examined in one test with two factorial analyses resulting in no-interaction (color × part and color × observation time), and each found no difference between colors. Observation time was examined in three tests with eight no-interaction analyses. In one test, none of the four analyses with 21, 7, 6, or 4 plant parts yielded differences among numbers of adults that were counted 2 h apart from 6 AM to 8 PM. The other two tests with observation times also found no differences. Apparently, *A. eugenii* does not discriminate between green or yellow fruit colors nor vary diurnally in its numbers. It occurs or makes oviposition and puncture marks on fruit equally at different observation times and on different fruit colors.

Across four field tests and three pepper cultivars for fruit characteristics, total numbers and weights of fruit were each highest for large and lowest for small fruit. “Hungarian” was the only cultivar differing among infested fruit numbers or sizes with higher numbers and weights of infested fruits generally found in large than in small fruits. This is similar to total fruit results, which found greater fruit numbers and weights in large than in small fruit not only for “Hungarian”, but also the other two cultivars. More importantly, among size classes within each cultivar, there were no differences in proportions of fruit numbers or of fruit weights that were infested. Seal and Bondari [13] similarly found that mean numbers of fruits infested with *A. eugenii* showed a significant positive correlation with numbers of fruit per plant. These differences suggest that similar proportions of different sizes of “Milta”, “Hungarian”, and “Cubanelle” fruit were infested by *A. eugenii.* According to Seal and Bondari [13], it infests similar proportions of different fruit numbers per plant as well. Hence, there appears to be no advantage in noting fruit-size differences when scouting to help locate *A. eugenii* adults.

## 4. Conclusions

Overall, *Capsicum chinense* “Habanero” was least susceptible, and *C. annuum* “SY” and “SR” were most susceptible to attack by *A. eugenii* based on counts of adults and marks from oviposition and feeding*.* However, “SY” and “SR” fruits tend to be numerically larger than “Habanero” and this may have affected the results. *Anthonomus eugenii* preferred the peduncle, calyx, and top of pepper fruits over the middle, bottom, leaves, or remainder of the flowers. There was no discrimination by *A. eugenii* between green or yellow fruit colors nor diurnal variation in its numbers. Based on adult counts, medium to extra-large fruits (≥1.5 cm long) were preferred over small fruits (<1.5 cm). However, based on fruit inspection, no differences were found between large and small fruits in proportions of fruit numbers or of fruit weights that were infested. Choice of pepper cultivar, preferred locations of adults, and their sites of feeding and oviposition on fruits may therefore be important in IPM programs which combat *A. eugenii* by reduced susceptibility, synchronous drop of infested fruits, or scouting. These results and suggested control measures may help in developing scouting programs and may best serve when fruits are small and applied insecticides may be preventative.

## Figures and Tables

**Table 1:** Test 1a: Numbers of *A. eugenii* adults found on different pepper cultivars and different parts of pepper fruit (from a cultivar × part factorial with interaction).

A. Comparing parts within cultivars^1^			B. Comparing cultivars within parts^1^			
	Mean (SD) ^2,3^	Mean (SD) ^2,3^			Mean (SD) ^2,3^	Mean (SD) ^2, 3^
Part	“Cayenne”	“Cherry”	Cultivar	Fruit L × W ^4^	Peduncle	Calyx
Peduncle	3.0 (3.1) ^a^	1.86 (1.70) ^ab^	“Cayenne”	8	3.0 (3.1) ^cd^	2.4 (1.6) ^b^
Calyx	2.4 (1.6) ^a^	2.36 (1.82) ^a^	“Cherry”	12	1.9 (1.7) ^d^	2.4 (1.8) ^b^
Top	1.6 (1.0) ^a^	1.14 (0.86) ^b^	“Cubanelle”	84	1.9 (1.7) ^d^	2.3 (1.0) ^b^
Middle	1.6 (1.7) ^a^	0.36 (0.63) ^c^	“Habanero”	15	0.6 (0.6) ^e^	0.8 (1.0) ^d^
Bottom	2.1 (2.4) ^a^	0.36 (0.63) ^c^	“Hungarian”	~40	3.1 (1.4) ^bc^	2.0 (1.5) ^bc^
*F,* df ^5^, *p*	NS	8.8; 4, 65; <0.0001	“Jalapeño”	18	0.7 (1.0) ^e^	1.1 (0.9) ^cd^
	**“Cubanelle”**	**“Habanero”**	“SR”	72	4.3 (2.2) ^ab^	8.3 (4.1) ^a^
Peduncle	1.9 (1.7) ^ab^	0.57 (0.65) ^a^	“SY”	~20	5.9 (4.0) ^a^	8.9 (4.5) ^a^
Calyx	2.3 (1.0) ^a^	0.79 (0.97) ^a^	*F,* df ^5^, *p*	-	9.5; 7, 104; <0.0001	27; 7, 104; <0.0001
Top	1.7 (1.3) ^ab^	0.57 (0.94) ^a^			**Top**	**Middle**
Middle	1.0 (1.5) ^bc^	0.36 (0.63) ^a^	“Cayenne”	8	1.6 (1.0) ^c^	1.6 (1.7) ^bc^
Bottom	0.8 (1.4) ^c^	0.29 (0.61) ^a^	“Cherry”	12	1.1 (0.9) ^cd^	0.4 (0.6) ^de^
*F,* df ^5^, *p*	4.3; 4, 65; 0.0040	NS	“Cubanelle”	84	1.7 (1.3) ^c^	1.0 (1.5) ^cde^
	**“Hungarian”**	**“Jalapeño”**	“Habanero”	15	0.6 (0.9) ^d^	0.4 (0.6) ^de^
Peduncle	3.1 (1.4) ^a^	0.71 (0.99) ^a^	“Hungarian”	~40	1.9 (1.7) ^c^	0.2 (0.4) ^e^
Calyx	2.0 (1.5) ^b^	1.14 (0.86) ^a^	“Jalapeño”	18	0.9 (1.0) ^cd^	1.1 (1.1) ^bcd^
Top	1.9 (1.7) ^b^	0.93 (1.00) ^a^	“SR”	72	8.9 (4.4) ^b^	2.1 (1.7) ^b^
Middle	0.2 (0.4) ^c^	1.14 (1.10) ^a^	“SY”	~20	19.9 (6.9) ^a^	6.6 (4.7) ^a^
Bottom	1.6 (1.3) ^b^	0.57 (0.85) ^a^	*F,* df ^5^, *p*	-	65; 7, 104; <0.0001	16; 7, 104; <0.0001
*F,* df ^5^, *p*	10.6; 4, 65; <0.0001	NS			**Bottom**	
	**“SR”**	**“SY”**	“Cayenne”	8	2.1 (2.4) ^b^	
Peduncle	4.3 (2.2) ^b^	5.9 (4.0) ^c^	“Cherry”	12	0.4 (0.6) ^d^	
Calyx	8.3 (4.1) ^a^	8.9 (4.5) ^b^	“Cubanelle”	84	0.8 (1.4) ^cd^	
Top	8.9 (4.4) ^a^	19.9 (6.9) ^a^	“Habanero”	15	0.3 (0.6) ^d^	
Middle	2.1 (1.7) ^c^	6.6 (4.7) ^bc^	“Hungarian”	~40	1.6 (1.3) ^bc^	
Bottom	2.7 (2.5) ^c^	5.8 (4.1) ^c^	“Jalapeño”	18	0.6 (0.9) ^d^	
*F,* df ^5^, *p*	15; 4, 65; <0.0001	14.6; 4, 65; <0.0001	“SR”	72	2.7 (2.5) ^b^	
			“SY”	~20	5.8 (4.1) ^a^	
			*F,* df ^5^, *p*	-	11; 7, 104; <0.0001	

^1^ Two-fruit total with 14 replications (one for each time of observation). ^2^ Data were transformed before statistical analysis, but only non-transformed means and standard deviations (SDs) are shown. ^3^ Means within a column followed by the same letter or no letter did not differ significantly based on analyses of variance followed by Waller-Duncan *K*-ratio *t*-tests (*p* ≥ 0.05). ^4^ Approximate fruit length × width (cm^2^) based on mean fruit sizes given in the Materials and Methods section. ^5^ df is shown for model, error, respectively.

**Table 2:** (Test 1b–d). Numbers of *A. eugenii* adults found on different pepper cultivars and different parts of pepper fruit. Based on factorials that yielded no interaction, which included cultivar × observation time with and without plant parts summed (A, B) and part × observation time with plant parts not summed (C).

Cultivar × Observation Time Comparing cultiVars with Observation Times Pooled ^1^				Part × Observation Time Comparing Parts with Observation Times Pooled ^1^	
Cultivar	Fruit L × W ^4^	A. Plant parts not summed ^2^	B. Plant parts summed ^2,3^	C. ^2^	
		Mean (SD) ^5,6^	Mean (SD) ^5,6^	Part	Mean (SD) ^5,6^
“Cayenne”	8	2.1 (2.1) ^c^	10.3 (6.3) ^c^	Peduncle	2.7 (2.7) ^b^
“Cherry”	12	1.2 (1.5) ^de^	5.3 (2.6) ^de^	Calyx	3.5 (3.8) ^ab^
“Cubanelle”	84	1.5 (1.5) ^cd^	7.6 (2.9) ^cd^	Top	4.6 (7.0) ^a^
“Habanero”	15	0.5 (0.8) ^f^	3.8 (3.5) ^e^	Middle	1.7 (2.8) ^c^
“Hungarian”	~40	1.8 (1.6) ^c^	8.1 (4.0) ^cd^	Bottom	1.8 (2.7) ^c^
“Jalapeño”	18	0.9 (1.0) ^ef^	5.3 (2.4) ^de^		
“SR”	72	5.3 (4.2) ^b^	25.7 (11.0) ^b^		
“SY”	~20	9.4 (7.2) ^a^	37.3 (18.9) ^a^		
*F,* df ^7^, *p*		67.6; 7, 552; <0.0001	42; 7, 152; <0.0001		11.4; 4, 555; <0.0001

^1^ There were no significant differences resulting from the factorials for cultivar × observation time comparing observation times with cultivars pooled (A, B) or part × observation time factorials involving observation times with plant parts pooled (C). ^2^ Replications. A. Plant parts not summed: 70, B. Plant parts summed: 20, C. 112. ^3^ For each cultivar, the results are based on the sum of five plant parts on each date (replication). ^4^ Approximate fruit length × width (cm^2^) using mean fruit sizes given in the Materials and Methods section. ^5^ Data were transformed before statistical analysis, but only non-transformed means and standard deviations (SDs) are shown. ^6^ Means within a column followed by the same letter or no letter did not differ significantly based on analyses of variance followed by Waller-Duncan *K*-ratio *t*-tests (*p* ≥ 0.05). ^7^ df is shown for model, error, respectively.

**Table 3:** Test 2: Numbers of puncture marks and oviposition plugs formed by adult *A. eugenii* and found on different pepper cultivars and different parts of pepper fruit. For each cultivar, three counts of puncture marks were taken on each part of two fruits resulting in 6 total replications per plant part per cultivar. Separate analyses were performed for groups involving individual parts and sums of parts per cultivar.

Cultivar × Part Factorial with Interaction							Sum of parts
A. Comparing parts within cultivars ^1^			B. Comparing cultivars within parts ^1^				C. One-way ANOVA ^1^
Cultivar ^2^	Part ^2^	Mean (SD) ^3,4^	Part^2^	Cultivar ^2^	Fruit L × W ^5^	Mean (SD) ^3,4^	Mean (SD) ^3,4^
“Cayenne”	Peduncle	0.33 (0.52) ^bc^	Peduncle	“Cayenne”	8	0.3 (0.5) ^c^	3.8 (1.9) ^b^
	Calyx	0.33 (0.52) ^bc^		“Cherry”	12	4.2 (1.5) ^a^	6.0 (2.0) ^b^
	Top	1.17 (0.98) ^ab^		“Cubanelle”	84	4.7 (3.4) ^a^	6.5 (4.4) ^b^
	Middle	0.00 (0.00) ^c^		“Habanero”	15	0.2 (0.4) ^c^	0.5 (0.6) ^c^
	Bottom	2.00 (1.41) ^a^		“Hungarian”	~40	1.2 (1.0) ^bc^	3.5 (2.1) ^b^
	*F,* df ^6^, *p*	6.1; 4, 25; 0.0014		“Jalapeño”	18	3.3 (4.5) ^ab^	6.0 (6.5) ^b^
“Cherry”	Peduncle	4.17 (1.47) ^a^		“SR”	72	4.2 (2.7) ^a^	5.8 (2.7) ^b^
	Calyx	1.67 (1.21) ^b^		“SY”	~20	3.5 (3.0) ^ab^	12.2 (6.8) ^a^
	Top	0.00 (0.00) ^c^		*F,* df ^6^, *p*		5.1; 7, 40; 0.0004	6.1; 7, 40; <0.0001
	Middle	0.17 (0.41) ^c^	Calyx	“Cayenne”	8	0.3 (0.5) ^cd^	
	Bottom	0.00 (0.00) ^c^		“Cherry”	12	1.7 (1.2) ^b^	
	*F,* df ^6^, *p*	33; 4, 25; <0.0001		“Cubanelle”	84	1.5 (0.8) ^b^	
“Cubanelle”	Peduncle	4.67 (3.44) ^a^		“Habanero”	15	0.0 (0.0) ^d^	
	Calyx	1.50 (0.84) ^b^		“Hungarian”	~40	0.8 (1.3) ^bcd^	
	Top	0.33 (0.52) ^c^		“Jalapeño”	18	1.5 (1.8) ^bc^	
	Middle	0.00 (0.00) ^c^		“SR”	72	1.3 (1.4) ^bc^	
	Bottom	0.00 (0.00) ^c^		“SY”	~20	4.3 (1.4) ^a^	
	*F,* df ^6^, *p*	19; 4, 25; <0.0001		*F,* df ^6^, *p*		6.9; 7, 40; <0.0001	
“SR”	Peduncle	4.17 (2.71) ^a^	Top	“Cayenne”	8	1.17 (0.98) ^ab^	
	Calyx	1.33 (1.37) ^b^		“Cherry”	12	0.00 (0.00) ^b^	
	Top	0.17 (0.41) ^c^		“Cubanelle”	84	0.33 (0.52) ^b^	
	Middle	0.17 (0.41) ^c^		“Habanero”	15	0.17 (0.41) ^b^	
	Bottom	0.00 (0.00) ^c^		“Hungarian”	~40	0.67 (1.21) ^ab^	
	*F,* df ^6^, *p*	11; 4, 25; <0.0001		“Jalapeño”	18	0.83 (0.75) ^ab^	
“SY”	Peduncle	3.50 (3.02) ^a^		“SR”	72	0.17 (0.41) ^b^	
	Calyx	4.33 (1.37) ^a^		“SY”	~20	3.33 (4.68) ^a^	
	Top	3.33 (4.68) ^ab^		*F,* df ^6^, *p*		2.3; 7, 40; 0.0426	
	Middle	0.17 (0.41) ^c^	Bottom	“Cayenne”	8	2.00 (1.41) ^a^	
	Bottom	0.83 (1.33) ^bc^		“Cherry”	12	0.00 (0.00) ^c^	
	*F,* df ^6^, *p*	4.8; 4, 25; 0.0055		“Cubanelle”	84	0.00 (0.00) ^c^	
				“Habanero”	15	0.17 (0.41) ^bc^	
				“Hungarian”	~40	0.83 (1.17) ^b^	
				“Jalapeño”	18	0.00 (0.00) ^c^	
				“SR”	72	0.00 (0.00) ^c^	
				“SY”	~20	0.83 (1.33) ^bc^	
				*F,* df ^6^, *p*		5.2; 7, 40; 0.0003	

^1^ Replications: 6. ^2^ There were no significant differences when comparing plant parts within cultivars for “Habanero”, “Hungarian”, or “Jalapeño” or when comparing cultivars within parts for the middle of fruit. ^3^ Data were transformed before statistical analysis, but only non-transformed means and standard deviations (SDs) are shown. ^4^ Means within a column followed by the same letter or no letter did not differ significantly based on analyses of variance followed by Waller-Duncan *K*-ratio *t*-tests (*p* ≥ 0.05). ^5^ Approximate fruit length × width (cm^2^) based on mean fruit sizes given in the Materials and Methods section. ^6^ df is shown for model, error, respectively.

**Table 4:** Test 3: Numbers of *A. eugenii* adults found on different parts of pepper fruit. Each of three factorial analyses (color × part, color × observation time, and part × observation time) yielded no interaction. Significant results from the one-way ANOVAs that followed are shown below.

Part ^3^	A. Color × Part	B. Part × Observation Time
	Comparing Parts (Colors Pooled) ^1,2^	Comparing Parts (Observation Times Pooled) ^1,2^
	Mean (SD) ^4,5^	Mean (SD) ^4,5^
Peduncle	2.2 (1.6) ^b^	2.2 (1.6) ^b^
Calyx	1.1 (0.9) ^c^	1.1 (0.9) ^c^
Top	4.4 (4.1) ^a^	4.4 (4.1) ^a^
Middle	0.7 (0.8) ^cd^	0.7 (0.8) ^cd^
Bottom	0.2 (0.4) ^d^	0.2 (0.4) ^d^
*F,* df ^6^, *p*	16.5; 4, 85; <0.0001	16.5; 4, 85; <0.0001

^1^ There were no significant differences comparing colors with plant parts pooled (A), comparing either colors with observation times pooled or observation times with colors pooled, or comparing observation times with plant parts pooled (B). ^2^ Test was conducted on a single day with 18 replications. ^3^ Top, middle, and bottom refer to visual approximations of the top, middle, and bottom thirds of fruit. ^4^ Data were transformed before statistical analysis, but only non-transformed means and standard deviations (SDs) are shown. ^5^ Means within a column followed by the same letter did not differ significantly based on analyses of variance followed by Waller-Duncan *K*-ratio *t*-tests (*p* ≥ 0.05). ^6^ df is shown for model, error, respectively.

**Table 5:** Test 4: Numbers of adult *A. eugenii* found at different locations on pepper plants during a single day. Results are shown for four factorial analyses each with 8 observation times as one factor, and the other factor was plant parts numbering 21 if ungrouped or 7, 6, or 4 if placed into groups.

**A. Twenty-one Parts ^1^**						
**Part ^2^**		**Mean (SD) ^3,4^**	**Part ^2^**		**Mean (SD) ^3,4^**	
Leaf		0.33 (0.64) ^ij^	Large fruit, peduncle		0.88 (1.75) ^ghij^	
Flower (not peduncle, calyx)		0.54 (0.93) ^hij^	Large fruit, calyx		1.88 (1.51) ^cd^	
Flower peduncle		0.46 (0.93) ^ij^	Large fruit, top		3.08 (2.32) ^ab^	
Flower calyx		0.63 (0.88) ^ghij^	Large fruit, middle		0.79 (1.32) ^ghij^	
Small fruit, peduncle		0.71 (1.12) ^ghij^	Large fruit, bottom		1.17 (1.46) ^efgh^	
Small fruit, calyx		0.67 (0.70) ^ghij^	Extra-large fruit, peduncle		1.17 (1.31) ^defg^	
Medium fruit, peduncle		1.25 (1.19) ^defg^	Extra-large fruit, calyx		1.42 (1.28) ^cdef^	
Medium fruit, calyx		1.46 (1.14) ^cde^	Extra-large fruit, top		3.96 (3.20) ^a^	
Medium fruit, top		2.08 (1.50) ^bc^	Extra-large fruit, middle		0.88 (1.19) ^fghi^	
Medium fruit, middle		1.54 (1.47) ^cde^	Extra-large fruit, bottom		0.33 (1.17) ^j^	
Medium fruit, bottom		1.67 (2.14) ^cdef^				
*F,* df ^5^, *p*					8.7; 20, 483; <0.0001	
**B. Seven parts ^1^**		**C. Six parts ^1^**		**D. Four fruit sizes ^1^**		
**Part^2^**	**Mean (SD) ^3,4^**	**Part ^2^**	**Mean (SD) ^3,4^**	**Fruit size^2^**		**Mean (SD) ^3,4^**
Leaf	0.33 (0.64) ^d^	Leaf	0.3 (0.6) ^d^	Small		2.5 (2.3) ^b^
Flower	0.54 (0.93) ^cd^	Flower	1.6 (1.8) ^c^	Medium		5.3 (3.4) ^a^
Peduncle	0.89 (1.30) ^bc^	Small fruit	3.9 (2.9) ^b^	Large		5.0 (4.0) ^a^
Calyx	1.21 (1.22) ^b^	Medium fruit	8.0 (4.7) ^a^	Extra-large		5.2 (4.2) ^a^
Fruit top	3.04 (2.53) ^a^	Large fruit	7.8 (5.2) ^a^			
Fruit middle	1.07 (1.36) ^b^	Extra-large fruit	7.8 (5.7) ^a^			
Fruit bottom	1.06 (1.71) ^bc^					
*F,* df ^5^, *p*	19; 6, 497; <0.0001		27; 5, 138; <0.0001			3.7; 3, 92; 0.0140

^1^ All four part × observation time factorial analyses yielded no-interaction results, and each follow-up ANOVA led to significant differences comparing parts, which are shown with the 8 observation times pooled. However, there were no significant differences among the 8 observation times when pooling the other factors: 4, 6, 7, or 21 parts and/or sizes. Sections A, C, and D each had 24 replications, while Section B had 24–120 replications. ^2^ Fruit size (length) included small (<1.5 cm), medium (1.5–3.4 cm), large (3.5–6.9 cm), and extra-large (≥7.0 cm). ^3^ Data were transformed before statistical analyses, but only non-transformed means and standard deviations (SDs) are shown. ^4^ Means within a column followed by the same letter or no letter did not differ significantly based on analyses of variance followed by Waller-Duncan *K*-ratio *t*-tests (*p* ≥ 0.05). ^5^ df is shown for model, error, respectively.

**Table 6:** Test 5: Numbers of *A. eugenii* larvae per plant found within fruits of selected pepper cultivars or cultivar groups in two commercial fields.

Cultivar or group	Fruit L × W ^1^	Field 1	Field 2
		Mean (SE) ^2^	Mean (SE) ^2^
“Hungarian wax”	~30	2.0 (0.7) ^b^	1.8 (0.4) ^cd^
“Habanero”	15	2.6 (1.2) ^ab^	1.2 (0.4) ^d^
“Jalapeño”	18	3.0 (1.2) ^ab^	3.0 (0.3) ^a^
Bell	~80	4.0 (0.8) ^a^	2.6 (0.5) ^ab^

^1^ Approximate fruit length × width (cm^2^) based on mean fruit sizes given in the Materials and Methods section. ^2^ Data were not transformed before analysis. Means within a column followed by the same letter or no letter did not differ significantly based on analyses of variance followed by Waller-Duncan *K*-ratio *t*-tests (*p* ≥ 0.05).

**Table 7:** Test 6: Comparison of different size (fruit length) classes based on numbers and weights of fruits. These included total fruits harvested and infested fruits.

Variables and Fruit Sizes		A ^1^ “Milta”	B ^1^ “Hungarian”	C ^1^ “Hungarian”	D ^1^ “Cubanelle”
Variable ^2,3^	Length	Mean (SD) ^4,5^	Mean (SD) ^4,5^	Mean (SD) ^4,5^	Mean (SD) ^4,5^
Total number of fruit	Small	18 (6) ^b^	4.9 (3.7) ^b^	2.6 (2.1) ^c^	1.0 (1.2) ^b^
	Medium	39 (12) ^a^	6.5 (2.9) ^b^	8.3 (5.7) ^b^	4.8 (3.0) ^a^
	Large	43 (12) ^a^	33.9 (17.7) ^a^	21.0 (11.4) ^a^	8.8 (2.4) ^a^
	*F,* df ^6^, *p*	17.7; 2, 24; <0.0001	23.6; 2, 21; <0.0001	19.7; 2, 21; <0.0001	13.4; 2, 9; 0.0020
Total fruit weight (g)	Small	49 (21) ^c^	9 (12) ^b^	8 (8) ^c^	10 (18) ^c^
	Medium	306 (74) ^b^	44 (21) ^b^	74 (61) ^b^	147 (100) ^b^
	Large	633 (203) ^a^	635 (434) ^a^	402 (233) ^a^	525 (121) ^a^
	*F,* df ^6^, *p*	103; 2, 24; <0.0001	39.3; 2, 20; <0.0001	46.1 2, 21; <0.0001	40.2; 2, 9; <0.0001
Number of infested fruit	Small	1.7 (1.8) ^a^	0.6 (0.9) ^b^	0.6 (0.7) ^a^	0 (0) ^a^
	Medium	2.7 (3.7) ^a^	1.9 (2.1) ^ab^	1.8 (1.8) ^a^	0.5 (1) ^a^
	Large	1.9 (2.2) ^a^	4.0 (3.6) ^a^	2.3 (1.8) ^a^	0.25 (0.5) ^a^
	*F,* df ^6^, *p*	NS	3.8; 2, 21; 0.0381	NS	NS
Weight of infested fruit (g)	Small	2 (3) ^a^	0.7 (1.0) ^b^	1.9 (3.3) ^b^	0.0 (0.0) ^a^
	Medium	14 (18) ^a^	10.4 (13.2) ^b^	12.4 (15.9) ^ab^	14.6 (29.1) ^a^
	Large	20 (20) ^a^	48.5 (47.1) ^a^	27.7 (23.9) ^a^	5.8 (11.5) ^a^
	*F,* df ^6^, *p*	NS	9.9; 2, 21; 0.0009	6.5; 2, 21; 0.0062	NS

^1^ Results of one-way ANOVAs each comparing three fruit-length classes (treatments) within each test for each variable. Replications: Test A (9), B (8), C (8), and D (4). Within each replication, each data point resulted from the sum of 5 subsamples (plants). ^2^ Numbers and weights of infested fruit are subtotals of the total numbers and weights of fruit, respectively, per 5-plant sample. ^3^ There were no significant differences among the three fruit sizes in any test (A–D) in the proportions of fruit numbers or weights that were infested. ^4^ Data were transformed before statistical analysis, but only non-transformed means and standard deviations (SDs) are shown. ^5^ Means within a column followed by the same letter or no letter did not differ significantly based on analyses of variance followed by Waller-Duncan *K*-ratio *t*-tests (*p* ≥ 0.05). ^6^ df is shown for model, error, respectively.
